# A Review of Wetland Remote Sensing

**DOI:** 10.3390/s17040777

**Published:** 2017-04-05

**Authors:** Meng Guo, Jing Li, Chunlei Sheng, Jiawei Xu, Li Wu

**Affiliations:** 1 School of Geographical Science, Northeast Normal University, Changchun 130024, China; xujw643@nenu.edu.cn; 2 Northeast Institute of Geography and Agricultural Ecology, Chinese Academy of Science, Changchun 130102, China; lijingsara@iga.ac.cn (J.L.); shengchunlei@iga.ac.cn (C.S.); 3 Remote Sensing Technique Centre, Heilongjiang Academy of Agricultural Science, Harbin 150086, China; aromawu@163.com

**Keywords:** wetland, remote sensing, optical sensor, radar, LiDAR

## Abstract

Wetlands are some of the most important ecosystems on Earth. They play a key role in alleviating floods and filtering polluted water and also provide habitats for many plants and animals. Wetlands also interact with climate change. Over the past 50 years, wetlands have been polluted and declined dramatically as land cover has changed in some regions. Remote sensing has been the most useful tool to acquire spatial and temporal information about wetlands. In this paper, seven types of sensors were reviewed: aerial photos coarse-resolution, medium-resolution, high-resolution, hyperspectral imagery, radar, and Light Detection and Ranging (LiDAR) data. This study also discusses the advantage of each sensor for wetland research. Wetland research themes reviewed in this paper include wetland classification, habitat or biodiversity, biomass estimation, plant leaf chemistry, water quality, mangrove forest, and sea level rise. This study also gives an overview of the methods used in wetland research such as supervised and unsupervised classification and decision tree and object-based classification. Finally, this paper provides some advice on future wetland remote sensing. To our knowledge, this paper is the most comprehensive and detailed review of wetland remote sensing and it will be a good reference for wetland researchers.

## 1. Introduction

Wetlands can be described as regions with low water levels, often near ground surface, which are covered by active plants during the growing season and water saturation period [1]. Wetlands are some of the most important and valuable ecosystems on Earth and are called “kidneys of the Earth”. There are two basic types of wetlands: natural and constructed [2]. Wetlands can improve water quality, protect shorelines, recharge groundwater, ease flood and drought severity, and provide unique habitats for many plants and animals. Scientists and government staff have paid increasing attention to wetlands to maintain the biodiversity of the aquatic system [2,3].

A wetland is a generalized concept including coastal wetlands, peatland, mangrove forests, estuarine wetlands, marshes, and other types of wetland. The coastal wetland functions as an important nutrient cycling capacity source to maintain water quality [4]. Peatlands play a key role in carbon cycle because they sequester carbon dioxide (CO_2_), the major greenhouse gas, from the atmosphere as peat; however, they also emis large quantities of CO_2_ and the second most important greenhouse gas, methane (CH_4_) [5]. Mangrove forests are important habitats for many fish and crustacean species and play important roles in coastal protection and water quality [6]. Despite the importance of wetlands, roughly 50% of wetlands have been lost since the 1900s [7]. Wetland-related studies are therefore of great importance to protect wetlands and mitigate climate change.

Remote sensing is the major source of spatial information about the Earth surface’s cover and constitution [4]. Different sensors capture abundant information regarding the Earth, which can be used by scientists that are interested in monitoring spatial information in a timely manner [8]. During the past five decades, remote sensing technology has been used in many wetland research areas such as: (1) land use/cover changes or mapping in wetland regions [4,9,10,11]; (2) carbon cycle and climate warming in wetland environments [5,12]; (3) release of carbon by peatland fires [13,14,15,16]; and (4) hydrology processes in wetlands [17,18,19]. Many wetland studies have used remote sensing technology and many researchers have reviewed wetland remote sensing. However, these reviews focus just on one type of wetland [20,21,22], on one place [23,24], on one type of sensor [25,26], or on one method [27]. Rundquist et al. [28] reviewed wetland remote sensing and addressed many issues of wetland identification, classification, change detection, and biomass. However, this paper was published in 2001, hence, it does not include the relevant publications of the past 15 years, a period in which remote sensing technology and wetland research have developed rapidly. A comprehensive review of wetland remote sensing has not been reported in recent years, so the objectives of this paper are to: (1) provide an overview of selected approaches and findings of wetland remote sensing; (2) highlight the different types of remote sensors in wetland remote sensing; and (3) provide advice for future wetland research.

## 2. Data and Methodology

The Science Citation Index Extended (SCIE) database from Web of Science [29] is the most comprehensive and frequently used data sources for reviewing high-quality publications and assessing scientific accomplishments so it was chosen for this study. The publications included journal papers and conference articles.

To categorize and manage all the papers of wetland remote sensing and also for convenience using in analyzing, this research built a database of “wetland remote sensing” First, a “wetland” database by searching for the following keywords was built: “wetland”, “peatland”, “bog”, “swamp”, “mangrove”, “marsh”, “mire”, “fen”, or “everglades” (date of search: 11 May 2016). In total, 93,894 papers were obtained. Then a thesaurus based on the Dictionary of Remote Sensing [30] was built to match the wetland database and established a database named “wetland remote sensing”. Intensive manual interpretation was necessary to obtain a complete set of papers of wetland research that used remote sensing. Finally, 5719 papers were selected for overview of wetland remote sensing. 

**Figure 1:**
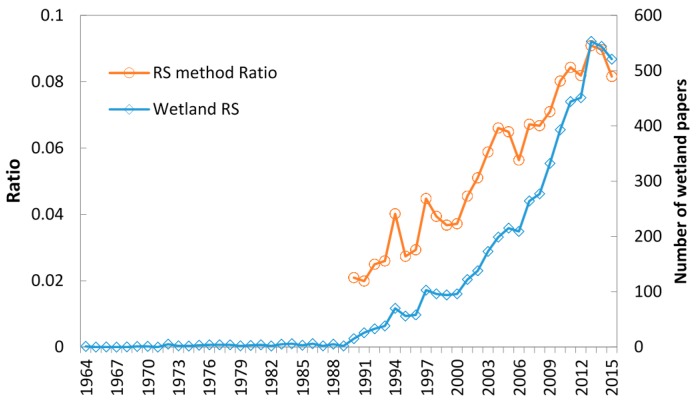
Number of wetland remote sensing publications and the ratio of wetland remote sensing to wetland research from 1964 to 2015.

Figure 1 shows the published wetland remote sensing papers and the ratio of wetland remote sensing to wetland research from 1964 to 2015.

## 3. Remote Sensing Techniques Used in Wetland Researches

In 1990, articles in Web of Science started to contain author keywords that could provide a description of a paper’s theme and concern and reveal an author’s research preferences. Therefore, keyword analysis could be used to explore research hotspots, directions and interests, and indicate scientific research trends and frontiers [31]. Table 1 lists different types of remote sensing sensors and their usage frequency for different study themes. More definitions and sensors can be found in the following sections. This study just reports the top 20 keywords for each sensor that appeared in the paper’s keywords. Medium-resolution images were mostly use in wetland research, followed by aerial photos, and hyperspectral images were used the least.

### 3.1. Aerial Photographs for Wetland Studies

Aerial photography allows the rapid collection of a large amount of data and a unique overview of an area. Aerial photography has been the principal remote sensing technology used to analyze ground surface events. The development of satellite remote sensing during the 1970s and 1980s has pushed the aerial-imagery analyses to a new level [20]. Only 17 papers were found before the 1990s that used aerial photography as the only information source to study wetland in the wetland remote sensing dataset. Johannessen [32] used aerial photographs of Nehalem Bay from 1939 and 1960 to compare the marsh boundaries in different aerial photographs with the U.S. Coast Survey chart from 1875 for seven estuaries. The paper’s idea was provoked by the circular pattern that appeared in the photographs; it was the earliest paper on wetland remote sensing. The author concluded that the appearance of abundant circular clumps of vegetation in the aerial photographs of estuarine mud flats in temperate regions was proof of the rapid expansion of marshes. Subsequently, Pestrong [33] used nine-lens multiband imagery within the range of 400–900 nm and other images in a tideland area of San Francisco Bay in the United States to develop a technique comparing their relative utility for specific geomorphic interpretations. The author argued that the nine-lens multiband imagery is excessive and can be mitigated to four bands (550–630 nm, near-infrared, Ektachrome color transparency, and Ektachrome-infrared transparency).

In the next few years, aerial photography was used in wetland mapping [34,35] and the visual interpretation method was developed. Wetland land cover classification and plant identification are often challenging due to the complexity of the wetland landscapes and the influence of biophysical variables, such as water levels, salt content, phenological vegetation variations, and density [36], but many studies have explored more methods to obtain accurate results. Scarpace et al. [35] used properly calibrated digitized aerial photography imagery to identify the wetland vegetation in Sheboygan Marsh of the United States by employing the visual interpretation method. To estimate the accuracy of the results, random points were selected; the accuracy was determined to be 56%–60%. The vegetation types could be clearly identified by color-infrared (CIR) aerial photographs because of the strongly reflecting plants in near-infrared wavebands to which CIR aerial photograph is sensitive [37]. Based on a comparison with other scales of aerial photographs in seven Indiana wetlands, Lovvorn and Kirkpatrick [38] found that 1:4800 scale CIR aerial photographs have potential for the accurate identification of all dominant species and that early September is an optimal time for species identification using color photographs because of the variation in fall senescence among species. Dale et al. [37] used CIR aerial photographs to identify the vegetation types in a subtropical coastal salt marsh in southeastern Queensland, Australia, by employing a polythetic divisive procedure. Based on a comparison of their results with unsupervised and supervised classifications, they found that the intrinsic visible variation on a photograph has an advantage over the other two classification methods. At last, four clearly separate salt marsh vegetation types were identified in the photos at macro-level; eight types were recognizable at micro-level. Ibrahim and Hashim [39] identified species groups of mangrove forest in 1:40,000-scale aerial photographs and determined three mangrove forest types with a 90% correct interpretation. Tiner [40] used high-altitude aerial photography with 1:40,000 to 1:130,000 scales as the primary data sources and visual stereoscopic photo interpretation for the identification, classification, and inventory of forested wetlands on a national basis in the United States. The author concluded that CIR aerial photography from the early spring is best for detecting deciduous forested wetlands in temperate regions.

Besides the visual interpretation techniques for wetland mapping, automated classification methods (unsupervised classification and supervised classification) were also carried out. Supervised classification was used for high-resolution multispectral imagery from the Compact Airborne Spectrographic Imager (CASI) to classify mangroves from Turks and Caicos. Six mangrove classes were identified with an overall accuracy of 78.2%. The authors concluded that CASI imagery can be used to assess mangrove areas with a greater level of detail and accuracy than with satellite sensors [41]. Furthermore, CIR photographs have been used to identify a special wetland plant. Everitt et al. [42] used supervised classification techniques to classify CIR photographs and distinguish wild taro along Rio Grande River in Southwest Texas and obtained classification results with higher accuracy (producer and user accuracies ranging from 83.3% to 100%). Unsupervised and supervised image analysis techniques were used for archived aerial CIR photographs to monitor black mangrove on South Texas Gulf Coast of the United States and provided accurate results [43,44]. Tuxen et al. [45] used supervised classification of CIR photographs to map the composition changes of the wetland vegetation in San Francisco Estuary, USA. The mapping accuracies ranged from 70% to 92%. Martin et al. [46] improved the image analysis for lower delta of Waikato River situated on west coast of the North Island of New Zealand using multispectral aerial photography and unsupervised classification with a high number of classes. Their results showed that the unsupervised classification accuracy can be improved with an increasing number of spectral classes, although it is likely that they did not achieve the upper accuracy limit with their maximum class size of 240 classes. The highest unsupervised classification accuracy achieved was a kappa value of 0.45 when 240 classes were used.

Aerial photography has an advantage in terms of spatial resolution and data acquisition time; however, wetland studies use these data typically in narrow coastal areas or along rivers because of the generally small cover areas. In most cases, aerial photographs are combined with other satellite images to study wetland on a regional or national scale. Matthews [47] mapped wood bison habitat in an area of 3383.5 km^2^ in the vicinity of the Mills and Mink lakes in the Northwest Territory of Canada using Landsat 5 Thematic Mapper (TM) as data sources and CIR photographs (1:20,000 scale) and ground surveys as reference data. Visual interpretation and automated classification methods were employed to classify the study region into ten habitat cover types, with an overall accuracy of 91%, and the two important winter forage habitats, sedge meadow and willow savanna, with an accuracy of 95% and 75%, respectively. Long and Skewes [48] used 1:50,000 panchromatic aerial photographs to check the mangrove map from Landsat TM satellite data in Southern Gulf of Carpentaria. Aerial video surveys have also been used to provide ground verification for mapping of wetland cover in Amazon Basin [49]. Aerial surveys can also be used as supplementary data for the land cover mapping in large areas based on coarse spatial resolution imagery (e.g., Advanced Very High Resolution Radiometer, AVHRR) [50].

White [51] used multi-sensors to capture the variation in spring wetland vegetation communities and surface expressions in Australian Great Artesian Basin. Airborne hyperspectral imagery was selected as reference data to discriminate the spring plant communities and surrounding substrate in detail. The combination of multispectral and multi-seasonal imagery (Quickbird, Korea Multi-Purpose Satellite-2 (KOMPSAT-2), aerial photographs) and Light Detection and Ranging (LiDAR) data were used by Rapinel et al. [52] to precisely map the distribution of wetland habitats in northeastern Brittany near the Mont-Saint-Michel Bay, France. They argued that the classification accuracy can be highly improved (overall accuracy = 86.5%) when combining LiDAR data and multispectral images. To overcome the signal saturation phenomena in regions of high aboveground biomass and multi-storied forest canopies using optical remote sensing data, Meng et al. [53] used texture indices derived from optical remote sensing data via Fourier-based textural ordination (FOTO) to map aboveground biomass in temperate forest of Northeastern China. Based on aerial photos with high spatial resolution, they calibrated the relationship between FOTO indices and field-derived biomass measurements and found that FOTO indices account for 88.3% of the variance in ground-based aboveground biomass.

Aerial photography techniques have been widely used in wetland studies because of their excellent advantages in terms of spatial resolution, cost and time, especially when satellite remote sensing techniques were in an early stage. Because of the challenges of data acquisition in large areas by flight, aerial photography has usually been used for the wetland mapping of small areas. After the launch of satellites, especially the launch of Landsat TM, aerial photography was mainly used for assessment of the classification procedures or biomass derived from lower-resolution remote sensing methods.

### 3.2. Review of Coarse Spatial Resolution Data for Wetland Studies

Coarse spatial resolution remote sensing data used in wetland studies includes Moderate Resolution Imaging Spectroradiometer (MODIS) and AVHRR data. MODIS instrument aboard NASA Aqua and Terra satellites provides nearly daily repeated coverage of the Earth’s surface with 36 spectral bands and a swath width of approximately 2330 km. Seven bands are specifically designed for land remote sensing with a spatial resolution of 250 m (bands 1–2) and 500 m (bands 3–7) [54].

Of the 5719 wetland remote sensing papers, 331 papers used MODIS data and 127 used AVHRR data. Because of the combination of spectral, temporal, and spatial resolution compared to other global sensors, MODIS has been used in a variety of studies such as water body mapping and inundation [55,56,57,58,59,60,61,62,63,64,65,66], water depth mapping [67], paddy rice planting area mapping [68,69], ecological disturbance analysis [70], wetland area monitoring [71,72,73], peatland mapping [54], and habitat mapping [74].

Satellite remote sensing data provide an effective and efficient tool for detecting water body areas and flood inundation extent on a large area. Because of the high temporal resolution and large coverage, MODIS has significant advantages for mapping the wetland extent and dynamics at a coarse spatial resolution [58]. Cai et al. [55] used MODIS data to map the water body areas of the Poyang Lake in China and obtained the lake surface area with an error of approximately 6.19%. They also analyzed the possibility of monitoring the height of the water surface using a radar altimeter. The surface water distribution was identified by Mizuochi et al. [56] using the modified normalized difference water index (MNDWI) of MODIS and normalized difference polarization index (NDPI) of the multi-frequency Advanced Microwave Scanning Radiometer Earth Observing System (AMSR-E) in North-Central Namibia. Takeuchi and Gonzalez [66] proposed a new model by combining 2 km MODIS NDWI and 16 km AMSR-E NDPI to predict daily land surface water coverage and obtained a result similar to that of MODIS. Using visible and infrared channels of MODIS data, Kaptué et al. [75] monitored the spatiotemporal variations of surface water from 2003 to 2011 in the Soudan-Sahel region of Africa and argued that MODIS could effectively characterize open water bodies larger than 50 ha. They also concluded that MODIS could be used to monitor the seasonal changes of surface water in semiarid regions. Based on time series MODIS data, Klein et al. [76] presented a new method using dynamic threshold techniques to map daily water body in global scale and concluded that the new method could obtain intra-annual changes of open water effectively. Prigent et al. [77] used AVHRR visible and near-infrared reflectance and the derived NDVI and other satellite data to estimate the area and monthly distribution of land surface water at global scale. They found that the declines of open water and the increases of population often occurred in the same region. Carroll et al. [78] produced a new water mask map globally at 250 m spatial resolution based on MODIS and radar based water body dataset. Their result improved the former water mask product at 1 km spatial resolution and could be freely download from http://landcover.org.

Ogilvie et al. [65] combined MNDWI and the normalized difference moisture index (NDMI) of MODIS imagery to monitor the flood areas. To monitor flood and estimate vegetation submerging in the Sundarban Delta, at the borders between India and Bangladesh, MODIS was used to evaluate the coverage fractions of bare soil, vegetated fields, and permanent water and AMSR-E was used to monitor the submerging vegetation [63]. Chen et al. [61] and Huang et al. [62] derived a flood inundation map from MODIS data in Murray-Darling Basin in Australia. Chen et al. [61] also used TM as the validation data and concluded that the accuracy of inundation mapping is mainly due to the spatial and spectral characteristics of MODIS imagery. Huang et al. [62] revealed spatial and temporal patterns of flood inundation in the study region. Dutta et al. [60] used the same method to derive inundation maps from MODIS daily imagery in Murrumbidgee Floodplain of Australia and then built a flood inundation model. Ordoyne and Friedl [58] used MODIS data to predict the surface inundation in Everglades of the United States and found that the inundation patterns correlate with the tasseled cap wetness index derived from MODIS. They also suggested that MODIS is a useful sensor for monitoring flood dynamics, especially in sparse tree wetlands. Kuenzer et al. [64] analyzed the dynamics of inundation using MODIS data in four river deltas of Asia and demonstrated that MODIS data have potential to depict distinct inundation patterns.

Compared to MODIS data, AVHRR data have been sparsely used for wetland studies because of the lower spectral resolution and coarse spatial resolution (8 by 8 km) of AVHRR normalized difference vegetation index (NDVI) data and also because of the natural fragmentation of wetlands [79,80,81,82,83]. Ewiii et al. [82] used NDVI from AVHRR images to monitor the damage of forested wetland by hurricanes in Louisiana, USA. They compared NDVI changes at three impacted sites with undamaged forest and confirmed the direct impact of hurricanes on the forested wetland. Ewiii et al. [83] used AVHRR NDVI and thematic maps classified based on Landsat TM to determine the relationship between forest type and hurricane impact severity and made conclusions useful for resource managers. AVHRR NDVI has also been used by Moreau et al. [81] to assess the spatial and temporal changes of biomass in wetland grasses of Bolivian Northern Altiplano; the authors found that NDVI is sensitive to green vegetation biomass. Time series of AVHRR NDVI have also been used to analyze the seasonal and annual changes of wetland and it was reported that NDVI time series can provide useful information for large area of wetlands [79]. Based on spectral mixture analysis, AVHRR images were used to estimate CH_4_ emissions from wetland in West Siberia. According to the CH_4_ emission rate of each wetland ecosystem from field observations and the fraction of each wetland ecosystem in each AVHRR pixel, Takeuchi et al. [80] determined the total and mean CH_4_ emission of the study region to be 9.46 × 10^6^ kg CH_4_ day^−1^ and 59.3 mg CH_4_ m^−2^ day^−1^, respectively.

### 3.3. Review of Medium Spatial Resolution Data for Wetland Studies

In this research, medium spatial resolution refers to the spatial resolution within the range of 4–30 m. In this paper, medium-resolution data mainly includes Landsat TM/Enhanced Thematic Mapper Plus (ETM+), Advanced Spaceborne Thermal Emission and Reflection Radiometer (ASTER), China & Brazil Earth Resource Satellite (CBERS), Systeme Probatoire D’Observation De La Terre (SPOT 1-4) and Advanced Land Observing Satellite (ALOS) Advanced visible and near infrared radiometer type 2 (AVNIR-2). Landsat data were used the most in wetland studies. Of the 5719 wetland research papers, 1259 papers used Landsat data. This research reviewed 95 papers of those 1259 papers based on six study themes (Table 2).

**Figure 2:**
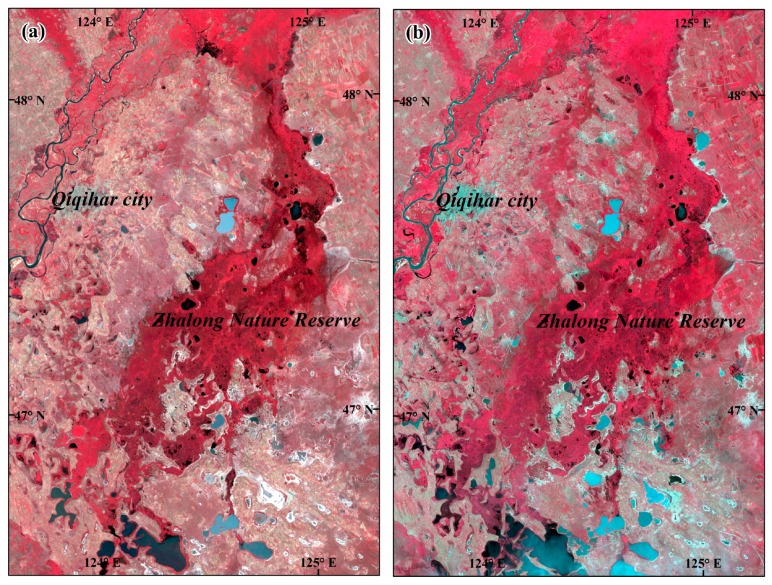

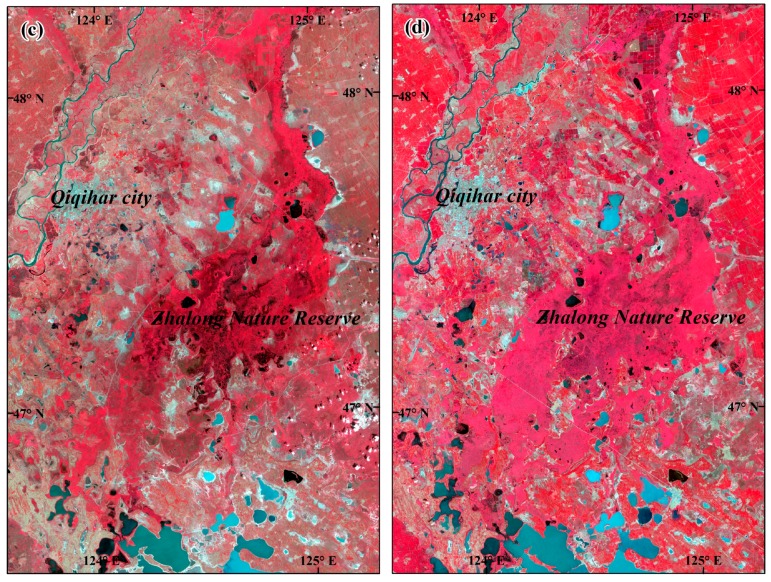
Zhalong Nature Reserve images (**a**) Landsat2 MSS on 1977183, RGB = 321; (**b**) Landsat5 TM on 1985194, RGB = 432; (**c**) Landsat 7 ETM+ on 2002185, RGB = 432 and (**d**) Landsat 8 OLI on 2016200, RGB = 543.

Landsat Multispectral Scanner (MSS) has been sparsely used in wetland studies because of its coarse resolution (80 m, recent distribution is resampled to 60 m); in many cases, MSS images were used together with TM and other sensors to prolong the time scale of wetland change detection [84,85,86]. Studies using TM data usually have higher accuracy of wetland mapping than MSS because of the improved spatial resolution and the increased number of optical bands [87]. Because of the recent launch (2013), Landsat 8 Operational Land Imager (OLI) data have not been used for many wetland studies. Figure 2 shows the Landsat images of Zhalong Nature Reserve in Qiqihar city of China.

#### 3.3.1. Mapping/Classification of Wetland

Wetlands have been threatened globally by human activities or climate change. These changes are reflected in wetland area shrinkage or vegetation cover or land cover changes. Understanding these changes could help to evaluate wetland ecosystems and provide information for environmental protection departments. Long-term change detection will enable researchers to better understand the trends and sudden changes of wetlands, analyze the dynamics, and protect wetlands. Mapping wetland areas, classifying the vegetation, and change detection are the most common research themes using Landsat data on a regional scale, not only because of the advantages in terms of spatial and temporal resolution but also because of the free data. Johnston and Barson [87] argued that the mid-infrared band of Landsat TM was useful for mapping wetland locations or ranges. Wetlands mapping or classification were mostly performed with the following aspects:

**Contribution of Landsat data to wetland vegetation classification.** Florenzano [90] used different Landsat TM and SPOT HRV data to map the geomorphology of a section of Taquari River in Brazil and concluded that TM can be used to create geomorphologic maps with a 1:100,000 scale. The author also emphasized that the precise relief units defined by TM and field survey data are quite important in mapping the geomorphology. Son et al. [85] used Landsat data from 1979 to 2013 to monitor the mangrove forest changes of Ca Mau Peninsula in South Vietnam. They found that the mangrove forest decreased by 16% over the past 34 years in this region and suggested that advanced management strategies should be proposed to protect mangrove ecosystem. Laba et al. [89] used MSS and TM data and assessed the dynamics of land cover changes in Yuna River watershed of the Dominican Republic to aid environmental planning and management. Gao [91] argued that Landsat TM (with high spectral resolution) obtained higher accuracies than SPOT High Resolution Visible (HRV) (with higher spatial resolution) when mapping mangrove because the higher spatial resolution increased the confusion of mangrove and non-mangrove. Multitemporal Landsat data can also be used to evaluate the magnitude of mangrove forest decline when scientific records are absent. Kovacs et al. [92] concluded that the mangrove forest of Teacapán-Agua Brava lagoon system in Mexico degraded notably, which coincides with reports of local elderly fishermen. Berlangarobles and Ruizluna [93] used six land cover types (mangrove, lagoon, salt marsh, dry forest, secondary succession, and agriculture) from Landsat data as direct indicators to detect landscape changes in the Majahual coastal system of Mexico. They found that farming activities were the main reason for landscape change. Ibharim et al. [94] argued that erosion, tree harvest, and farming activities are the main threats related to mangrove degradation. Jia et al. [100] and Long et al. [101] used Landsat data to map the distribution of mangrove forests in China and Philippines, respectively, advance the management, and protect mangroves. Qin et al. [157] used Landsat ETM+ and OLI data to map paddy rice planting area and obtained precise results with 97.3% accuracy. Rapinel et al. [178] found that Landsat 8 OLI images can be used to map plant communities in coastal marshlands with higher accuracy.

**Development of a new method to map and monitor wetlands.** Johnston and Barson [87] compared different classification methods and found that density slicing of the mid-infrared band of Landsat TM is a suitable method for mapping the location and range of wetlands. Jensen et al. [88] used an image with adequate field survey information as standard data; images from other years were normalized to the radiometric characteristics of the standard image to monitor the aquatic macrophyte change within Florida Everglades Water Conservation Area. Petropoulos et al. [95] proposed a semi-automatic classification method based on support vector machines (SVMs) to quantify the deposition and erosion in two Mediterranean wetland deltas using Landsat TM data. NDVI and Land Surface Water Index (LSWI) derived from Landsat images were used by Dong et al. [98] to map wetland areas and provided satisfactory results. Borro et al. [99] proposed a new method for mapping shallow lakes using NDVI derived from Landsat data. After comparing their results with other studies, they found that the new method can increase the accuracy of lake boundary and also considers the dynamics of shallow lakes. Jia et al. [100] mapped mangrove areas in China using an object-oriented classification method applied to Landsat data and obtained accurate results. Ausseil et al. [158] presented a rapid method for wetland mapping on an extended scale using Landsat ETM+ images. It just took one and a half months to map and rank the wetland of Manawatu-Wanganui region in New Zealand. Based on Landsat ETM+ images, Chiu and Couloigner [159] proposed a modified Fuzzy C-Means (FCM) classifier to identify potential wetland areas and obtained better results than the standard FCM classifier.

#### 3.3.2. Flooding/Inundation

Floodplains are transitional zones of aquatic and terrestrial environments [109]. Wetlands are the water storages of flood plains [108]. The location and extent of inundation need to be understood to manage rivers or flood plains.

Landsat data have been used for flood mapping or to build flood models in wetland flood studies [102,104,106,110,113,180]. Robinove [180] pioneered the use of Landsat images when mapping floods in Queensland, Australia. He proposed that the dark areas in the Landsat images are wet soil but not flooded regions. NDWI and sum of bands 4, 5, and 7 derived from Landsat TM and ETM+ images were used by Thomas et al. [104] to map the inundation in Macquarie Marshes in New South Wales. They checked the accuracy of the inundation map results using aerial photography and found that the overall accuracies reached 93%–95%. Based on Landsat TM and other land cover data, Todhunter and Rundquist [106] reported a 53% increase of the terminal lake area and 426% increase of the area of rural wetland ponds in Devils Lake Basin in North Dakota of the United States; the increased areas cause floods. Frazier and Page [108] used Landsat TM images before and after the flood to describe the relationship between flow regulation and inundation in floodplain wetlands. The results show that river regulation could reduce the duration and frequency of inundation. Polo and Gonzálezdugo [113] monitored the flooded area change and vegetation development after the dismantling of the dam near the mouth of San Pedro River Marsh in Spain using the Spectral Mixture Model (SMM). They concluded that Landsat data were suitable to characterize the two aspects mentioned above both with respect to spatial and temporal resolution. Using millions of Landsat images, Pekel et al. [181] quantified the global changes of surface water between 1984 and 2015 and found almost 90,000 km^2^ permanent surface water disappeared and new 184,000 km^2^ formed. They also analyzed the reasons of these changes in different regions and found that long-term droughts and human activities are the most important influence factors. Feng et al. [182] produced a global dataset of surface water at 30 m resolution based on 8756 Landsat data and found the inland water area is 3,650,723 km^2^. Overton [107] built an inundation model based on GIS, RS, and a hydrological model. Landsat TM images were used to monitor the extent of flood inundation. It was reported that the model identifies flood inundation at a large area and lower cost. Profeti and Macintosh [105] used Landsat TM and European Remote Sensing (ERS) Synthetic Aperture Radar (SAR) to model the hydrological behavior and flood; Landsat TM was used to estimate soil water content. Wang [102] proposed an efficient and economical approach to map the flooded area in a coastal floodplain. They used the reflectance features of water and land from Landsat images before and after the flood to monitor the extent of the flood and obtained satisfactory results. Carle et al. [111] used Landsat TM and other images to assess the impact of the 2011 Mississippi River flood on Wax Lake Delta. Their results indicated that the infrequent and large floods are important for the maintenance of river deltas. Using a time series of Landsat images, Mueller et al. [103] described a method to observe water in Australia. Decision trees and logistic regression were used in their research to distinguish persistent and ephemeral water.

#### 3.3.3. Habitat/Biodiversity

Wetlands are considered a wildlife habitat. Some types of endangered birds are restricted to specialized wetland habitats. Habitat loss and degradation due to climate change and human activities greatly threaten waterfowls. Climate change, such as frequent drought, could cause the shrinkage of wetland area; human activities, such as wetland reclamation and water conservancy engineering, are thought to be the reasons for habitat loss and degradation and threaten the survival of waterfowls [127]. As a medium-resolution sensor, Landsat data have been considered to be an effective method in habitat research of wildlife in wetlands at a large area, especially because of time scales >30 years.

Tulbure et al. [129] used Landsat data to derive surface water bodies and then identify the “step stone” of water-dependent organisms to maintain landscape connectivity. Toral et al. [123] used Landsat data to identify rice paddy stages and mapped the habitat availability of water birds in rice fields. Discriminant function analysis was used for Landsat images and different paddy field stages; the habitats of different species of water birds were obtained. Their results are important for water bird conservation, especially in regions with small areas of natural wetland habitat. Jiang et al. [127] evaluated the habitat for Siberian cranes of the Poyang Lake wetland in China using Landsat data and found that the suitable water level is 12 m. Their results have practical significance for controlling the water level of the Poyang Lake Dam. Delgado and Marín [126] used NDVI derived from Landsat data to assess the habitat of the black-snacked swan. Wang et al. [125] assessed the habitat quality of the red-crowned crane in the Yellow River Delta Nature Reserve of China using Landsat data. They concluded that human activity should be minimized to improve the habitat quality for red-crowned crane. Human activities disturb the ecosystem, which will affect the survival of bird species. Maclean et al. [163] examined the relationships between harvesting, burning, and habitat fragmentation of swamps and six bird species and found a positive correlation. With the increase of swamp areas, all six bird species increased; however, but no correlation was observed depending between the shape and proximity of swamps and bird species. Landsat and land cover data can provide estimates for water bird habitats in wetlands; the dynamics and distribution of water bird habitats are essential for conservation [122].

A reliable wetland habitat map is required by ecologists and conservationists; however, different methods applied to the Landsat data could lead to results with different accuracies. Poulin et al. [161] reported that the simple maximum likelihood (ML) function is suitable to map rare wetland habitat and the weighted maximum likelihood (WML) function is more suited for common habitats. To guide the conservation of endangered shorebirds in the future, The population of shorebirds and their suitable habitat should be understood. Many scholars modeled species–habitat relationships based on wildlife observation and habitat data. Long et al. [165] mapped a suitability habitat for a type of shorebird using Landsat data and then estimated the number of shorebirds. Slattery and Alisauskas [119] used Landsat TM data to monitor the snow geese habitat and estimated the spatial distribution and habitat use in flock and individual scales of snow geese. Giardino et al. [179] mapped the coastal and submerged habitat using Landsat 8 OLI and in situ data to study the principal element of biodiversity. Harvey and Hill [116] and Duffett et al. [117] modeled the nest habitat of saltwater crocodiles using Landsat TM, SPOT, and aerial photography and Matthews [47] assessed bison habitat using Landsat TM data.

#### 3.3.4. Biomass/Carbon Stock

Wetland biomass can be used to reflect the status of the wetland ecosystem and biomass estimates can provide basic information about wetland planning and utilization. Peregon et al. [170] classified Western Siberia into 30 landscapes based on a field survey and remote sensing data (Landsat ETM+, SPOT) and then used aerial photography to evaluate the ratio of micro-landscapes in wetlands. They evaluated the biomass of wetland in Western Siberia. Tan et al. [167] calculated the biomass of the Poyang Wetland in China using a linear regression model between the biomass derived from a field survey and the vegetation index obtained from Landsat ETM+ data.

Partial least squares regression was used to select the appropriate spectral features of field survey and satellite reflectance to model the aboveground biomass of some marsh species. It was found that Landsat ETM+ and Hyperion infrared bands have slight advantages for the biomass estimation of wetlands [173]. The underground biomass of wetlands was also estimated for peatland [174]. A hybrid model based on the aboveground biomass and N content of leaves was proposed to estimate the underground biomass; 76% of the variation below the ground could be explained.

Mangrove forest biomass has been focused on in many studies because of the higher biomass content and the challenges estimating the biomass. To estimate the biomass of mangroves, the vertical information is important because trunks and branches contain the vast majority of biomass. Radar remote sensing has potential for the estimate because of the penetration capability. Fatoyinbo et al. [169] used Landsat ETM+ and Shuttle Radar Topography Mission (SRTM) data to estimate the biomass of mangroves in Mozambique. Fatoyinbo and Simard [172] added LiDAR canopy height estimates and also derived three-dimensional (3-D) structure and biomass maps of mangroves in Africa. Some researchers argued that Radarsat images acquire the mangrove biomass with higher accuracy than Landsat TM images because of the higher resolution and side-looking geometry [133].

Peatland plays a key role in carbon balancing and it acts as source or sink based on the status of near-surface water tables. The accurate assessment of the carbon stock of peatland has significant effects on understanding the carbon cycle. Draper et al. [135] used remote sensing images together with field survey data to evaluate the carbon stock of peatland in Amazonia. Crichton et al. [175] used Landsat data to estimate the carbon balance in peatland of the UK and reported results that are in good agreement with the finer-scale estimates. Many scientists also estimated the carbon storage in mangroves [136,137,138]. All these studies were significant for land cover management and even for carbon cycle.

#### 3.3.5. Water Quality in the Wetland

Landsat data used in water quality monitoring can be grouped into two categories, one uses the reflectance changes of the Landsat bands to monitor water quality [86,140,141,142,144,145,146] and the other one employs Landsat data to identify land cover types and then determine the relationship with water quality of field survey [143,176].

The transport of sediment influences the type of biology and geomorphic processes. Mertes et al. [86] used spectral mixture analysis of Landsat MSS and TM data to estimate the suspended sediment concentrations in Amazon River wetland; the endmembers used in the spectral mixture analysis were obtained in the laboratory. Mccullough et al. [145] used Landsat TM bands 1 and 3 to estimate water clarity. Lagos et al. [140] argued that the increase and decrease of the water reflectance in the visible and near-infrared bands, respectively, were the results of turbid water and the decrease of water plants. Kumar et al. [144] used the reflectance of Landsat TM bands 2, 3, and 4 to regress water pollution parameters. A neural network model that uses Landsat reflectance data were proposed by Chebud et al. [141] to quantify water quality parameters. Landsat TM images could also be used to map water quality and then identify the potential risk [142].

Choi and Han [143] and Costantini et al. [176] used Landsat data to identify the land cover types around water and then evaluated water quality to determine how land cover changes affect environment.

#### 3.3.6. Trace Gases from Wetland

Greenhouse gases (GHGs) have become a hot topic in recent years because of global warming. The concentration of CH_4_, which is the second most important GHG following CO_2_, has doubled over the past 200 years [148], with a recent increase rate of ~1% [152]. In total, 15% of the global warming has been thought to be contributed by CH_4_ [152]. Many researchers pay attention to the CH_4_ fluxes from wetlands, which are considered to be the largest natural source of CH_4_ [147].

Chamber-based trace gas flux measurements were commonly used to monitor trace gases from soil which results just on behalf of the characteristic of points (usually within 1 m^2^). Sometimes people are more concerned about the trace gas flux on a large area. It is challenging to extrapolate the trace gas fluxes from a quadrat scale to landscape or regional scale. Many studies used chambers or enclosures to measure trace gas fluxes for each land cover type and used Landsat data to classify the land cover types of the study region. They determined trace gas flux on a regional scale using the sum of the trace gas fluxes of each land cover type [147,148,149,152,155,177].

### 3.4. Review of High Spatial Resolution Optical Data for Wetland Studies

This paper defined the high spatial resolution image as images with the spatial resolution <4 m, mainly including data from SPOT-5, IKONOS, Quickbird, WorldView, and GeoEye. Compared with medium-resolution and hyperspectral images, images with high spatial resolution have more geometry and texture information on the surface features and can be used to identify ground features easily. However, they usually have few channels. In this study, 73 papers that used high spatial resolution sensors were reviewed. All these papers were divided into three categories: (1) proposed a new method to classify wetlands based on high spatial resolution images; (2) used higher spatial resolution to obtain wetland map with higher accuracy; and (3) mangrove forest studies.

#### 3.4.1. Improvement of Classification Method

High spatial resolution images provide detailed information about ground surface and are a milestone of remote sensing. However, the high spatial resolution images also lead to new challenge with respect to classification.

The traditional pixel-based, supervised, and unsupervised classifications derive ground surface information based on spectral information of pixels; however, the structure and texture of ground features and the relationships between contiguous pixels are not considered. With the increasing spatial resolution of images, too many land cover types and differences between them appear in the images and it is difficult to identify the ground feature using pixel-based classification. Because of the huge amount of pixels of the high spatial resolution images, high-performance computer are needed and the classification will take too much time.

Object-based classification is a new technology that was developed for high spatial resolution images. It can integrate multi-source remote sensing data or remote sensing and GIS data. The principle of object-based classification is to group objects that have similar features, such as a similar pixel shape, color, or texture, and classify them based on the object features.

Seventeen of the 73 research papers used the object-based classification. Hubert-Moy et al. [183] and Hassan et al. [184] used the object-based method for high-resolution images (SPOT or WorldView-2) to map and analyze wetland and found that object-based method can clearly identify land cover types. To overcome the shortcomings of medium- or coarse-resolution images when identifying wetland fringe and species, Heumann [185] used object-based image analysis and WorldView-2 sensor to map mangrove species. He obtained a satisfactory mangrove species map (overall accuracy >94%) but no accurate mangrove fringe map because of the similar spectral features between mangroves and associate species. Rokitnicki-Wojcik and Wei [186] used IKONOS images to map wetland habitat. Wang et al. [9] compared three classification methods for IKONOS images to map mangroves, pixel-based and object-based classification and the integration of the pixel- and object-based classification. They verified that the classification result with the best accuracy was obtained using the integration of the pixel- and object-based classification. Object-based and per-pixel maximum likelihood classifications were also performed on WorldView-2 images by Lantz and Wang [187] to obtain accurate classification results.

New methods were also proposed based on high spatial resolution. Stavrakoudisa et al. [188] proposed a Boosted Genetic Fuzzy Classifier (BGFC) to map land covers using IKONOS images. Based on the comparison with the traditional classification method, they found that the new method processes multi-dimensional feature spaces more effectively. Keramitsoglou et al. [189] used a Kernel-based reclassification algorithm for WorldView-2 images over wetland to overcome the limitations of the transitional classification method with respect to identifying boundaries of habitat and the barriers in combination with field survey and remote sensing data. Kamal et al. [190] and Kamal et al. [191] used the semi-variogram tool to identify mangrove features based on WorldView-2 images. The semi-variogram is a geostatistical method used in remote sensing; it examines the variation of the pixel values within a series of distances. Semi-variograms were used to determine the spatial structure and distribution of feature sizes in the mangrove environment. To increase the accuracy of the urban wetland identification using remote sensing images, Xu and Ji [192] developed a new classification method by integrating the pixel-based classification with a knowledge-based algorithm and concluded that the new approach effectively increased the classification accuracy. Skurikhin et al. [193] presented a semi-automated approach to recognize and hierarchically partition water-body regions with WorldView-2 imagery. Fagherazzi et al. [194] proposed an automatic method, which identifies the location and area of the channel bed from SPOT images to extract tidal channel network.

#### 3.4.2. Obtain Fine-Accuracy Maps

High spatial resolution images are of advantage for the identification of wetland boundary and species. Many scholars used high spatial resolution images in wetland studies to improve the research accuracy. Midwood and Chow-Fraser [195] used two stages of IKONOS images to compare the changes of four wetland plant communities. Leempoel et al. [196] used GeoEye-1 and other sensors to assess the mangrove forest decline in Gaoqiao of China.

SPOT, IKONOS, and WorldView-2 data were used to generate coastal and marine habitat map [197], evaluate both the extent and consequences of floods [198], describe shoreline changes [199], classify land cover [200,201,202,203], and map mangroves [185,204].

#### 3.4.3. Mangrove Forest Study

Mangroves are salt-tolerant forest species that live in intertidal zones, which provide important ecosystem characteristics and services [205]. However, mangrove forest areas have notably declined in the past 50 years due to anthropic destruction [206]. Some authors argue that mangrove forest will be lost soon, without appropriate and effective actions [205,207]. Mangrove research has been a research frontier in recent years. Twenty of the 73 research papers that study wetlands using high spatial resolution images focus on mangrove forest. Three main categories were discussed in these 20 papers: (1) classification or mapping of mangroves [9,185,208,209,210]; (2) mangrove evolution or degradation [196,204,211,212,213]; and (3) mapping of mangrove species [205,206,210,214,215,216]:

**Classification or mapping of mangroves.** Conchedda et al. [209] used object-based classification and SPOT data to map the land cover in the mangrove ecosystem in Senegal and reported clear changes between 1986 and 2006 with an overall classification accuracy of 86%; a 94% overall classification accuracy was obtained when object-based analysis, support vector machine classification and WorldView-2 images were used [185]. Gao [208] used SPOT images to map mangroves and discussed the reasons for the different classification accuracies.

**Mangrove evolution or degradation.** The decline of the mangrove forest has been verified globally. The decline is due to human activities and its rate is different in different regions. Tong et al. [211] used SPOT images to assess how shrimp aquaculture impacts mangrove ecosystem. They classified mangrove ecosystem into five ecologically distinct parts and analyzed the possible links to shrimp aquaculture for some regions of Vietnam. They concluded that, from 1965 to 2002, approximately 30% of mangrove ecosystem has been lost and more than 30% of the present mangroves are replanted monospecific species. During the second Indochina war, many mangrove forests have been destroyed; at present, these regions were reforested with monocultures. Landscape heterogeneity is essential for ecosystem survival and lightning is the main natural disturbance of mangroves. To estimate the capability of lightning to increase the heterogeneity, Vogt et al. [212] used SPOT images to detect and quantify the disturbance and heterogeneity of mangrove forests. They proposed the importance of small disturbances in maintaining the biodiversity of mangrove forests. The forest degradation can be reflected by the green leaf, which can be calculated from remote sensing data. Kovacs and Flores [213] determined the relationship between leaf area index (LAI) from field surveys and the vegetation index derived from satellite imagery (IKONOS) in disturbed mangrove forests; they established a close relationship, which indicates that IKONOS can be used as a robust tool in monitoring mangrove forest degradation.

**Mapping of mangrove species.** Mangrove forests often comprise different species depending on the distance of water edge, which fulfill different ecological functions and have a different ability to resist risk. It is urgent to identify mangrove forest structure to better understand basic mangrove information and guide conservation and restoration activities. Because of spatial resolution limitations, conventional images have been rarely used for mangrove species identification. To obtain an accurate mangrove species map, many scholars explored the use of high spatial resolution images. Neukermans et al. [205] employed Quickbird images to identify four dominant mangrove species and obtained satisfactory results with an overall accuracy of 72%. Wang et al. [206] used two IKONOS images from different seasons (February and May 2004) to identify mangrove species. Wang et al. [214] compared the mangrove species maps from IKONOS and Quickbird images and found that both sensors have the capacity to classify mangrove species.

### 3.5. Review of Hyperspectral Data for Wetland Studies

Hyperspectral data are obtained using imaging spectrometers and provide complete and continuous spectral information with a large number of narrow bands (<10 nm) within the range of 0.38–2.5 µm. Hyperspectral imagery comprises dozens to hundreds of narrow bands and a continuous spectral profile for each pixel. It greatly increases the detailed information on vegetation and has been widely used in wetland research because of the complex vegetation composition. Satellite, airspace, and hand-held hyperspectral data have been successfully used in wetland mapping, wetland species identification, plant leaf chemistry research, wetland soil property analysis, and other themes.

#### 3.5.1. Wetland Mapping/Classification

Wetlands are characterized by a complex and lush vegetation composition and usually it is a challenge for traditional optical sensors to map them. Hyperspectral sensors have been widely used for wetland classification because of the advantages with respect to the band number and continuous reflectance values for the ground vegetation. Belluco et al. [217] used multi-spatial and hyperspectral data to map tidal marshes, which have complex patterns of geomorphic and ecological features and found that better classifications can be obtained from hyperspectral data compared with multispectral data. They also concluded that spatial resolution has more important effects on the accuracy of tidal marsh classification than spectral resolution. Rosso et al. [218] evaluated the usefulness of hyperspectral imagery from AVIRIS (Airborne Visible and Infrared Imaging Spectrometer, with 224 bands ranging from 380 to 2500 nm and 20 m spatial resolution) in a marsh structure study of San Francisco Bay, California. Spectral mixture analysis (SMA) and multiple endmember spectral mixture analysis (MESMA) were applied to AVIRIS image. The authors concluded that both methods are suitable to map the main components of the marsh.

Factors that alter the ecological function of wetlands are considered to be degradations. To evaluate this, Schmid et al. [219] applied a spectral unmixing approach to the hyperspectral and multispectral data in a semi-arid wetland of Central Spain and obtained successful results. They observed rapid vegetation changes due to anthropogenic influences.

Hyperspectral images from the Compact High Resolution Imaging Spectrometer (CHRIS)/Project for On-Board Autonomy (PROBA) mode 2 with 18 bands were used by Karaman et al. [220] to examine seasonal changes of the water-covered area in Denizli, Turkey, and determined the most useful band combination separating water from land (bands 2, 6, 15, and 18). Bands 6 and 18 and bands 2 and 18 were the band combinations most appropriate to measure NDWI. Space- and airborne hyperspectral images and other optical sensor images were used by Barducci et al. [1] to monitor wetland characteristics and assess biogeochemical features in the coastal zone of San Rossore Natural Park in Italy. They concluded that images with <10 m spatial resolution and <5 nm spectral resolution are needed to better manage the wet areas in a coastal region influenced by human activities.

To better understand wetland conditions, Kumar and Sinha [221] used high spatial resolution data from Quickbird and hyperspectral data from Hyperion to map the salt marsh vegetation communities of Micalo Island in Australia; they found that a better accuracy (overall accuracy = 71.1%) was obtained using Hyperion compared with Quickbird (overall accuracy = 59.4%).

Yang et al. [222] mapped black mangroves on South Texas Gulf Coast of the United States using airborne AISA+ hyperspectral imaging. Minimum noise fractions (MNF) were used to eliminate the redundancy band of AISA+ and four classification approaches were applied to the noise-reduced hyperspectral imagery and band-eliminated MNF imagery. The results showed that the maximum likelihood method applied to the band-eliminated MNF imagery provides a black mangrove map with excellent accuracy.

Zhang [223] combined the hyperspectral image from AVIRIS and LiDAR data to map the vegetation in Everglades in Florida. He observed that LiDAR improved the map accuracy compared (overall accuracy = 86%) with the application of hyperspectral images alone (overall accuracy = 76%).

#### 3.5.2. Wetland Species Identification

Hyperspectral sensors provide tens to hundreds of narrow bands, which provide detailed wetland vegetation information. They greatly improve the wetland vegetation type identification [20]. Mangrove forest is one of the most important ecosystems because of its role in protecting coastal environment. Mangrove species identification provides basic information on wetland inventory and vegetation community changes.

Wang and Sousa [224] measured the spectral reflectance of mangrove leaves in the laboratory using a high-resolution spectrometer and concluded that the most useful bands for mangrove species identification were the bands at 780, 790, 800, 1480, 1530, and 1550 nm. Kamal and Phinn [225] used a CASI-2 hyperspectral dataset with 30 bands and 4 m spatial resolution to map mangrove species at the mouth of Brisbane River in Australia. The results from pixel-based (spectral angle mapper, SAM, and linear spectral unmixing, LSU) and object-based methods were compared and it was concluded that the object-based approach is the best method for mangrove species identification. Kumar et al. [226] used an Earth Observing-1 Hyperion image with 196 bands ranging from 357 to 2576 nm to identify the floristic mangrove composition in Bhitarkanika National Park, India, and determined five mangrove classes with satisfactory accuracy.

Hyperspectral data ranging from 400 to 2500 nm were used to discriminate 46 vegetation species in tropical wetlands by Prospere et al. [227]. Subsequent to comparisons with other classification methods, the authors found that the generalized linear model fitted with elastic net penalties (GLMnet) was the best method to discern the plant types in wetlands.

Species invasion is a serious type of ecosystem disturbance, which decreases biodiversity and threatens the resident species with extinction. To manage the invasive species, the amount and distribution of them must be understood, which is a challenge using traditional optical sensors. Hyperspectral images provide sufficient spectral detail to resolve this challenge. Artigas and Yang [228] used Airborne Imaging Spectroradiometer for Applications (AISA) to map the marsh species in New Jersey Meadowlands of the United States and concluded that *Phragmites* have a similar reflectance across the growing season, which might be used to map their distribution. Andrew and Ustin [229] used HyMap imagery with 128 bands and 3 m spatial resolution to map the invasive plants of *Lepidium latifolium* in three wetlands of California. Classification and regression tree models (CART) and mixture-tuned match filter (MTMF) and spectral physiological indexes were used in their study; satisfactory results were obtained at two wetland sites, with ~90% accuracy; however, the results for other sites are characterized by poor accuracy. The reasons for success and failure were analyzed.

#### 3.5.3. Leaf Chemistry of Wetland Vegetation

Water quality in many wetlands has declined progressively over the past several decades because of the increasing usage of recycled water in wetlands and the inflow of nutrients from agricultural and urban areas. Wetland water eutrophication with high concentrations of chlorophyll occurred after pollution [230]. Siciliano et al. [230] first collected nutrients and other water quality data at 18 water sampling stations and then fertilized two sampling stations of them and measured the changes of the tissue composition of C (carbon), H (hydrogen), and N (nitrogen) and the spectral responses of leaves and canopy. They used HyMap hyperspectral imagery with 126 bands ranging from 440 to 2500 nm at 2.5–3.0 m spatial resolution to assess wetland ecosystem and found positive relationships between the N levels and plant responses for both plot scale and landscape scale. Significant correlations were also observed between water nutrients and the photochemical reflectance index (PRI) and derivative chlorophyll index (DCI). They concluded that hyperspectral data can be used to detect nutrient concentrations in wetlands.

Nitrogen is one of the most important components in plant leaves and its change can be monitored using hyperspectral data. Zhang et al. [231] examined the relationship between leaf N concentration and laboratory hyperspectral data corresponding to two mangrove species under healthy, poor, and dwarf conditions. Two models, artificial neural network (ANN) and stepwise multiple linear regression (SMLR), were employed to predict N concentrations based on multiple wavebands; the former model predicted N concentration more accurately than the latter.

Flores-De-Santiago et al. [232] tested 35 vegetation indices (VI) derived from in situ hyperspectral measurements (450–1000 nm) to estimate the leaf chlorophyll content based on 360 leaf samples during both the dry and rainy seasons. The results showed that VIs derived during the dry season obtained better estimates of lead chlorophyll than that of the rainy season and the index of R740/R720 was the best predictor of the mangrove leaf chlorophyll content.

To model leaf chlorophyll and N concentration using remote sensing data, Kalacska et al. [233] examined the relationship between spectral reflectance and leaf chlorophyll and N concentration for 19 species in peatland during the growing season. A model that comprised a continuous wavelet transform and a neural network scalable from leaf to airborne hyperspectral imagery was proposed in their study and obtained satisfactory results (with a R^2^ ranging from 0.8 to 0.9 for both chlorophyll and nitrogen). Guo and Guo [234] selected *Phragmites australis* and *Typha angustifolia* as targets and used correlation analysis and stepwise regression analysis applied to spectral reflectance in visible domain to estimate the chlorophyll and N contents in wetland. They found that the range of 700 to 750 nm was the most useful spectral range to estimate biochemical parameters. They also concluded that the difference of the data types and biological differences greatly influence the results.

#### 3.5.4. Wetland Soil

Soil quantity, especially the content of N and organic matter, will directly affect the health of the vegetation, which can be characterized by hyperspectral imagery. To examine the possibility of using hyperspectral reflectance of vegetation canopy to inverse the soil electrical conductivity, Li et al. [235] selected *Crypsis schoenoides* in Central California-managed wetland as study subject and used three indices derived from field spectrometer hyperspectral data, individual narrow-band reflectance, first-derivative reflectance, and narrow-band normalized difference spectral index (NDSI), to determine the soil electrical conductivity using an univariate regression model. These indices provided valid results based on the high correlation coefficient (R^2^) of 0.56, 0.59, and 0.57 for individual narrow-band reflectance, first-derivative reflectance, and narrow-band NDSI, respectively. Anne et al. [236] argued that Hyperion hyperspectral images have the potential to predict the soil properties that indicate the ecosystem nutrients and organic matter storage. They collected soil samples of the mangrove forest and salt marsh on western coast of Central Florida. The single-band and two-band indexes and partial least squares (PLS) statistical models for Hyperion hyperspectral image were tested; the best statistical models were derived from the two-band index and PLS.

#### 3.5.5. Other Themes

Mangroves are the most important plants along coastlines, estuaries, lagoons, and rivers because of their salt-tolerance. However, the higher salt content will also affect the mangrove health. Song et al. [237] used the photochemical reflectance index (PRI) derived from hyperspectral data collected in the field as proxy for the photosynthetic rate to monitor the growth of red (*Rhizophora mangle*) and white (*Laguncularia racemosa*) mangroves under different salinity and determined strong correlations for both species.

Wong and Fung [238] used hyperspectral imagery from EO-1 Hyperion and radar data from Environmental Satellite (ENVISAT)/Advanced Synthetic Aperture Radar (ASAR) supplemented with in situ field surveys to explore their capability in monitoring the biophysical conditions of mangrove stands through LAI estimation. They concluded that radar data alone cannot effectively map the mangrove LAI; however, the combination of hyperspectral and radar data leads to an improvement.

Ong et al. [239] monitored the anthropogenic iron oxide dust on the vegetation using airborne hyperspectral data and found that the dust can be observed within 2 km from the source and that rainfall reduces the dust load. Cole et al. [240] modeled the vegetation abundance of peatland using airborne hyperspectral data and field survey data. They proposed an empirical model to predict the vegetation abundance of plant functional types (PFTs) using partial least squares regression (PLSR) and obtained satisfactory results.

### 3.6. Review of Radar Data for Wetland Studies

Spaceborne optical data have been widely used since the 1970s to study vegetation and environment. The launch of Landsat and Terra/Aqua pushed the use of optical sensors to a new level. However, the biggest weakness of optical data is the presence of clouds and haze. In some regions, clouds and rain last for a long time because of the monsoon cycle; this period is sometimes also important for the plant growth. Optical images usually fail to monitor vegetation types within wetlands because the dense vegetation cover leads to signal saturation [241]. Radar data have several advantages for the acquisition of ground information over optical data because radar is capable to penetrate clouds. The use of radar data started a new era of land surface remote sensing. But limitations of radar lie in the limited availability of the data, lack of regular and long time series of coverage, cost of data, and complexity in analysis.

Because of the strength of penetrating vegetation canopy and acquiring ground information all day without the limitation of clouds, radar data, such as ALOS Phased Array L-band Synthetic Aperture Radar (PALSAR), European Remote Sensing (ERS-1), RadarSAT, ASAR, Japanese Earth Resources Satellite 1 (JERS-1), AIRSAR, and TerraSAR-X, are uniquely suited to identify and monitor changes of soil moisture, flooding, and aboveground biomass in wetlands [242].

#### 3.6.1. Wetland Mapping

Maillard et al. [243] used Radarsat-1 and ASTER data to delineate and characterize palm swamps to understand the extent and category of the palm swamps, which are thought to be the only water source of wetland ecosystem during dry seasons. They used Radarsat-1 and the combination of Radarsat-1 and ASTER to classify the palm swamps. They found that the SAR-based classification yields better results when the images were obtained during or after rain seasons with a low incidence angle but ASTER data have advantages for classifying the main vegetation forms. The optical images are limited to cloud-free conditions and might fail to precisely describe the extent of saturated areas and the water cycles in wetlands. To overcome this, Marechal et al. [244] employed time series RADARSAT-2 data to monitor the seasonal changes of wetlands using the proposed supervised PolSAR (Polarimetric SAR) segmentation methods. The results showed that RADARSAT-2 data could be used to delineate water feature in detail. Touzi et al. [245] analyzed the capacity of RADARSAT-2 for wetland mapping and monitoring using Touzi’s decomposition method and they emphasized the importance of the phase of the major backscatter type of wetland. Their results verified the potential of the RADARSAT-2 data for wetland classification. Wetland PFTs could be an indicator of wetland state and also reflect environmental changes and disturbances. Morandeira et al. [241] estimated the use of C-Band Polarimetric SAR from RADARSAT-2 to map the wetland areas covered by different PFTs in floodplain. Their results showed that the complex polarimetric information has better accuracy than the multi-polarization stack. They also concluded that the C-Band shallow incidence angle scene shows better results for herbaceous PFTs than the steep incidence angle scene.

Bartsch et al. [246] discussed the potential of ENVISAT ASAR data for global wetland monitoring. The results showed that the ASAR Global mode is capable of obtaining the area and dynamics of wetland, even at coarse spatial resolution. Betbeder et al. [247] evaluated the potential of TerraSAR-X imagery in monitoring the distribution of wetland plants to prevent flooded areas and found that TerraSAR-X data can be used to create a precise wetland herbaceous map. They also argued that polarimetric parameters are quite important for vegetation classification. Ferreira-Ferreira et al. [248] used multitemporal PALSAR L-band radar imagery to derive vegetation structure and flood areas and duration. The results showed that SAR remote sensing has the potential to provide accurate information on vegetation and inundation and unique insights into wetland ecosystem functions. Romshoo et al. [249] used a time series of L-band JERS-1 SAR data to monitor the deforestation in a peatland swamp forest and found that the cultivated crop in the deforested regions fails to grow because of the unsuitable water and soil chemistry. They also reported the potential of L-band SAR for flood detection in peatland. Kushwaha et al. [250] used various filters to suppress the random noise of ERS-1 SAR data and concluded that the Gaussian Sigma-2 filter with a 5 × 5 pixels kernel was the most efficient method in coastal wetland studies.

#### 3.6.2. Emergency Mapping

A flood is a natural disturbance of wetlands and information is needed immediately to create a destruction map and rescue plan [251]. Radar images are used for this purpose instead of optical images because of clouds and rain. In fact, SAR images have been proven useful for monitoring floods because of the all-weather, all-time capabilities in obtaining ground information. The backscattered signal of flooded and non-flooded vegetation in wetland regions is different; based on this, Grings et al. [252] developed a method to predict the flooded marsh.

Radarsat-1 SAR data are an effective data sources for detecting flood extent in coastal marshes. The backscattered signal of SAR data have a positive correlation with water level [253]. Kiage et al. [253] also revealed that marsh flood can be best described by the changes of two flood images. It has been verified that the combinations of multitemporal ALOS PALSAR and RADARSAT-2 data are efficient in mapping land cover and flood patterns in wetland regions [254]. Airborne SAR images were used by Horritt et al. [255] to map the flooded vegetation in a salt marsh using two techniques: C-band backscatter-enhanced and L-band HH-VV phase differences. Their results showed that both methods improve the mapping of the emergent vegetation and can be used to reduce the errors of flood maps. Furthermore, RADARSAT-2 has different beam modes for different purposes. Brisco et al. [256] compared the results of water level changes and flooded vegetation from different modes of RADARSAT-2 and found that the wide-swath high-resolution modes are appropriate for wetland monitoring and water level estimates because of the appropriate coherence and backscatter. Zhao et al. [257] revealed the land cover characteristics of Erguna Floodplain in China using six polarimetric RADARSAT-2 images; they reported that multitemporal image classification can describe vegetation cover with better accuracy. They also concluded that the intensity of the backscatter signal is important for land cover mapping. JERS-1 SAR and Envisat ASAR data were also efficient for the classificaton of flooded wetland and flood monitoring [252,258,259]. TerraSAR-X, ERS-1/ERS-2, Radarsat-1, and Shuttle Imaging Radar with Payload C (SIR-C) data have been used to reveal the water level changes in wetlands of different countries [260,261,262].

O’Grady and Leblanc [263] explored mapped flood areas nearly in real time using C-band radar data. Based on the comparison of notably different backscatter responses of the two large flood cases, they concluded that the backscatter signal is affected by many factors, such as the water level, vegetation density, and biomass, and shows complex changes. Hidayat et al. [264] used PALSAR data to derive the flood extent and flood occurrence information in tropical wetlands. They incorporated water level measurements in lakes and peatlands in their study and revealed that the flood under forest canopy corresponds to high backscatter values.

#### 3.6.3. Biomass Estimates

Vegetation biomass in wetlands is an important indicator of carbon sequestration of wetlands. Wetland biomass controls the carbon that could potentially be released to the atmosphere. Therefore, biomass estimates in wetlands are essential for understanding the carbon cycle of the wetland ecosystem. Many scientists have used optical remote sensing data for this purpose. Radar data also have the potential to estimate vegetation biomass in wetlands, not only because of the cloud-penetrating capability of the radar, but also because radar is particularly sensitive to the vegetation canopy over an underlying water surface [265]. Moreau and Toan [265] mapped the biomass of *totora* reeds and *bofedal* in water-saturated Andean grasslands using ERS SAR data to protect this ecosystem from overgrazing. They found that the backscatter signal of ERS SAR was sensitive to the humid and dry biomass of reeds and grasslands and their biomass maps were useful for the livestock management in the study region. Hamdan et al. [266] and Darmawan et al. [267] found that L-band ALOS PALSAR can be used to estimate the aboveground biomass because of the correlation between HH and HV backscatter signals and the aboveground biomass. The RADARSAT-2 data were used by Shen et al. [268] to inverse the biomass of the Poyang Lake in China based on backscatter characteristics of the C-band.

#### 3.6.4. Wildfires and Other Disturbances

As a natural disturbance, wildfires usually occur in moorland and sometimes deep in peat. Peatland fires release CO_2_ and CO into the atmosphere and polluted water source. It is essential to understand peatland fire information, such as location, burned area, and last occurrence, to protect the nearby environment. Millin-Chalabi et al. [269] proved that SAR can detect fire scar boundaries accurately after a precipitation event and can also be used to monitor peatland fire persistence.

Radar data have also been used in other research fields. Taft et al. [270] found that RADARSAT can be effectively used to identify the habitat of shorebirds in agricultural land. Forsberg et al. [271] used JERS-1 L-band SAR images to explore how tectonic faults affect the distribution of wetland.

### 3.7. Review of LiDAR Data for Wetland Studies

Light Detection and Ranging (LiDAR) is a remote sensing technology used to monitor the Earth surface. It measures the distance between sensor and target object using a pulsed laser. LiDAR can generate three-dimensional information about ground and its surface and is therefore used to derive the Digital Elevation Model (DEM).

#### 3.7.1. Forest Height

LiDAR data have been successfully used in mapping the elevation of forest ecosystems using the advantage of obtaining an accurate DEM. For full waveform LiDAR, the first returned signal is thought to be reflected from the canopy and the last returned signal is from the ground surface [272]. Wetland researches based on LiDAR are a challenge because of the difficulties of LiDAR pulses in penetrating the thick vegetation cover [273].

Richardson et al. [274] proposed a new method to quantify the geomorphic forms of forested wetland using LiDAR data; they report the feasibility of LiDAR data with respect to this purpose. They also presented two quantitative indices: lagg width index (LWI) and lateral slope index (LSI). The former index is used to quantify the width of the mixed areas between the wetland and upland and the latter one is used to measure the shape of the wetland ground surface. The gaps in the forest generated by disturbance play a key role in the forest ecosystem recovery. Zhang [275] used LiDAR to derive the elevation difference between the ground surface and canopy and then identified the gaps in the mangrove forest. In their study, which was successful, alternative sequential filtering and black top-hat mathematical morphological transformation were used to determine the forest gaps. Boehm et al. [276] used two temporal LiDAR datasets from August 2007 and August 2011 to monitor peat swamp forest changes. They used LiDAR to measure the height of forest and compared the results with field survey. They reported an average regrowth of 1.9 m and a peat decrease of 18 cm within the four years.

#### 3.7.2. Sea Level Rise

Sea level rise has caused many disasters along coastal zones such as flood, saltwater intrusion, and storm surges. Sea level rise is also a critical factor for the stability of salt marshes. Because of the advantages of LiDAR data in deriving accurate ground elevations, sea level rise and related research are also key research themes using LiDAR data. Zhang [277] analyzed the DEM derived from LiDAR data and concluded that the sea level rose by 1.5 m by 2100 in South Florida. To estimate the threat of sea level rise to salt meadows and to analyze how the elevation affected the meadow plant species richness and salt tolerance, Moeslund et al. [278] proposed a model to quantify the sea level rise associated with salt meadow vegetation and concluded that fine-resolution topography based on LiDAR data is required.

#### 3.7.3. Combination with Other Sensors

As mentioned above, many scholars have mapped and monitored wetlands using optical remote sensing images, radar data, and LiDAR data separately and obtained satisfactory results. The combination of these three types of remote sensing data was also assessed for different wetland types.

Pavri et al. [279] combined ASTER and LiDAR data to map the structure of coastal wetlands and obtained accurate results. LiDAR plays an irreplaceable role in the determination of the elevation and structure of landscapes, whereas other techniques have difficulties to identify vegetation and water. Huang et al. [280] monitored wetland inundation using Landsat and LiDAR data. In their paper, LiDAR data were first used to calculate the inundation ratio of a 30 m Landsat image; subsequently, the relationship between the inundation ratio and the surface reflectance was determined based on Landsat data. At last, they built an effective inundation model based on Landsat data. Elevation is a key factor affecting the spatial distribution of mangroves. The use of a digital terrain model (DTM) derived from LiDAR data may increase the mangrove map accuracy. Chadwick [281] found that the overall accuracy increased by 7.1% when combination of IKONOS and DTM data was used compared with using the IKONOS data alone. Rapinel et al. [52] compared the wetland classification accuracy by using LiDAR data in combination with multi-spectral and multi-seasonal images and high spatial resolution images alone and also found that the accuracy clearly increased when using the combination of methods. Chust et al. [282] used the combination of LiDAR data with multi-spectral images for mapping wetland habitats and came to similar conclusions. Allen et al. [283] reported that multi-date SAR and the combination of multi-date SAR and multi-date LiDAR both obtained a classification with higher accuracy; no clearly difference was found between the two approaches.

## 4. Future Wetland Remote Sensing Studies

### 4.1. Multi-Source Integrations for Wetland Classification

Wetland classification using remote sensing data has been performed for more than 50 years. Results with different accuracies were obtained by different researchers. Data used include the earlier photography data, medium-resolution images, high-resolution images, hyperspectral images, radar and LiDAR data. Many authors used remote sensing data combined with field survey data to carry out many wetland studies. Also many authors used higher spatial resolution data to enhance the accuracy of wetland researches. Some researchers suggest that the integration of different data sources has the potential to increase the classification accuracy, especially when using the integration of optical images with radar or LiDAR data. Furthermore, some scholars argue that the use of multi-season remote sensing data could increase the classification accuracy. It could conclude that multi-source integration should be the trend of future wetland remote sensing.

### 4.2. Wetland Remote Sensing on a Large Area

Previous studies show that most wetland remote sensing research focuses on the regional or national scales; only very few projects consider the continental or global scale. It was known that some wetland change effects, such as on climate change, might be visible on a large area. Because of huge workload, it is a hard task to map wetlands on a large area using medium- or high-resolution remote sensing images. With the development of computer technology, high performance computing offers many opportunities to realize large area moderate and fine resolution classification in the near future. With the maturity of the decomposition of mixed pixel technology, coarse-resolution could be used to globally map wetlands. NOAA and MODIS data are the most commonly used coarse-resolution data and have the potential to map global wetland changes when using high-resolution images to derive wetland endmembers. Many vegetation indices that derived from MODIS, such as NDVI, Enhanced Vegetation Index (EVI), have been successfully used in researches of land cover changes. These indices with time series could also be used in wetland researches in large area, especially in the topics of wetland degradation. New methods should also be explored to map wetland on a large area with higher accuracy.

### 4.3. Scale Effect Study Using Remote Sensing Data

The scale effect of remote sensing data means that using images with different spatial resolutions will lead to classification results with different accuracies. Previous research mainly used one type of remote sensing image to study wetland and higher-resolution or field survey data as the training and accuracy check data. However, what spatial and spectral resolutions of remote sensing data are suitable for a special study?

The scale effect is a true phenomenon; however, only few studies focused on it in the past. Of course, higher spatial resolution data could obtain detail information, but researchers must pay more money in getting data and pay more time in data processing. Sometimes, for some studies, especially in large area, sciences just want to know the trend of wetland, result accuracy isn’t the most important. For a specific research area and research theme, there must be a most fitting data resolution to analyze wetland features based on the cost of the data acquisition, time spend on the data processing, and result accuracy. Studies should pay more attention to the scale effect in the future.

### 4.4. More Research Group Exchange

A research team usually focuses on one object and study regions close to the location of the research institute. A research team usually publishes a series of gradually advancing papers, which is a good reference for other studies. Researchers of different research groups usually have different research backgrounds such as remote sensing, environment, and ecology. The exchange and cooperation between them could spark new ideas. New ideas may solve more environmental and ecological problems in the future.

Information sharing is needed in future studies, especially data and methods sharing between researchers who focus on the same study regions. To do that, the development of data sharing platform such as cloud database or data sharing website is needed.

Workshops including several research groups, especially those from different countries, should be held more frequently. In the workshops, scientists could learn about current research themes of other scholars, international problems at the frontier of science, and new methods to solve a scientific problem. In general, a workshop or international conference is the place to learn from each other.

### 4.5. New Data and Methods Used

With the development of remote sensing technology, more satellites will launch in the future and some military satellite data, such as Sentinel series of satellites, could also be used in the next few years. The remote sensing data with increased spatial and spectral resolution will be used in wetland researches and scientists will have more available data. Furthermore, new methods that used for wetland identification, water quality, biodiversity and other study themes should be proposed in the future. In the next few years, new methods that could increase result accuracy are needed.

## 5. Summary and Conclusions

This paper provides an overview of wetland remote sensing of the past half century. More than 250 papers were reviewed with respect to seven categories: aerial photographs, coarse-resolution images, medium-resolution images, high-resolution images, hyperspectral images, radar data, and LiDAR data.

Aerial photographs were the earliest used remote sensing data in wetland studies. Because of the lack of aerial photographs and the restrictions of remote sensing technology, wetland remote sensing papers were not abundant and the research topics mainly focused on wetland boundary analysis and wetland mapping. In recent years, aerial photography has been used as a type of high spatial resolution imagery to classify wetlands, identify wetland species, and test the accuracy of other classifications or maps.

Coarse-resolution data of MODIS and AVHRR were used in many wetland studies. Many researchers used the combination of MODIS and medium- or high-resolution data to monitor wetland changes. MODIS data have a relatively low spatial resolution but cover large areas and have a higher spectral resolution; therefore, many scientists used MODIS data to identify wetlands. Many indices, such as NDVI, NDWI, NDMI, and NDPI, of MODIS and AVHRR data were also commonly used in wetland studies.

Medium-resolution images were used the most, not only for wetland research but also for other themes. Medium-resolution images have been used in many research fields such as for wetland maps or classifications, flooding or inundation in wetlands, and habitat and biomass estimates. It has been confirmed that Landsat TM/ETM+/OLI data are suitable for many research fields; they are the most used remote sensing data in wetland research. The free use of Landsat data is a benefit.

High spatial resolution images have been widely used for wetland species identification and wetland classification and are also suitable for mangrove forest research. Because of the high price of high-resolution images, they were mainly used in small study areas, to explore new methods, or verify the wetland map accuracy. Many researchers have verified that high spatial resolution images have the potential to improve the classification accuracy.

Hyperspectral images can be used for wetland classification, species identification, and especially to determine wetland vegetation leaf chlorophyll and nutrient concentration because of the advantages in band number and spectral resolution.

When optical sensors are not feasible, radar data have been widely used in the field because of the penetration capability**.** Radar data have been successfully used for flood extent mapping and to identify the flooded vegetation in flood plains. Radar data have been successfully used for biomass estimates and wildfire mapping.

LiDAR data can be used to derive 3-D maps of the ground surface and elevation information such as the height of forests or the water level. Some scientists have verified that the combination of LiDAR and optical images has the potential to improve the wetland classification accuracy.

This study also proposed wetland remote sensing in future studies. Such as multi-source data integration, enlarge the study area of wetland researches, consider the scale effect of wetland researches and enhance groups’ exchange. In conclusion, this paper will provide a good reference for wetland research using remote sensing.

## Figures and Tables

**Table 1:** Remote sensing sensor usage frequency for different themes.

Theme	Aerial	Coarse-Resolution	Medium-Resolution	High-Resolution	Hyper-Spectral	Radar	LiDAR
Vegetation	456	332	710	166	209	387	156
Land use/cover change	184	189	594	118	59	197	75
Classification	200	117	664	170	147	291	89
Habitat	221	34	199	46	38	62	59
Conservation	141	37	218	44	22	63	36
Climate change	85	73	126	16	9	70	39
Biomass	59	56	141	37	51	121	58
Restoration	120	27	100	17	16	37	33
Hydrology	74	26	96	9	11	98	37
Biodiversity	54	29	115	15	10	37	32
Modeling	40	42	72	17	10	57	44
Flooding	57	47	117	11	5	126	21
Disturbance	70	21	65	8	11	22	12
Change detection	29	25	149	27	8	43	8
Sea level rise	77	14	49	9	15	28	52
Water quality	30	12	55	11	8	26	7
Soil moisture	7	29	40	11	9	83	12
Ecosystem services	20	12	64	9	8	15	21
Sedimentation	48	4	30	4	1	24	14
Evapotranspiration	6	45	29	2	2	10	6
Σ	1978	1171	3633	747	649	1797	811

**Table 2:** Wetland studies using Landsat data for seven study themes.

Sensor	Mapping/Classification	Flooding	Habitat/Biodiversity	Biomass/Carbon Stock	Water Quality	Trace Gases
MSS	[84,85,88]				[86]	
TM	[48,85,87,89,90,91,92,93,94,95,96,97,98,99,100,101]	[102,103,104,105,106,107,108,109,110,111,112,113,114]	[47,115,116,117,118,119,120,121,122,123,124,125,126,127,128,129,130]	[131,132,133,134,135,136,137,138]	[86,139,140,141,142,143,144,145,146]	[147,148,149,150,151,152,153,154,155,156]
ETM+	[85,94,96,98,99,100,101,157,158,159,160]	[103,104]	[121,122,123,124,127,129,161,162,163,164,165,166]	[167,168,169,170,171,172,173,174,175]	[140,143,176]	[177]
OLI	[68,85,157,178]		[179]			
